# International Collaboration to Ensure Equitable Access to Vaccines for COVID‐19: The ACT‐Accelerator and the COVAX Facility

**DOI:** 10.1111/1468-0009.12503

**Published:** 2021-03-02

**Authors:** MARK ECCLESTON‐TURNER, HARRY UPTON

**Affiliations:** ^1^ Keele University

## Abstract

**Policy Points**

- Equitable access to a COVID‐19 vaccine in all countries remains a key policy objective, but experience of previous pandemics suggests access will be limited in developing countries, despite the rapid development of three successful vaccine candidates.
- The COVAX Facility seeks to address this important issue, but the prevalence of vaccine nationalism threatens to limit the ability of the facility to meet both its funding targets and its ambitious goals for vaccine procurement.
- A failure to adequately address the underlying lack of infrastructure in developing countries threatens to further limit the success of the COVAX Facility.

**Context:**

Significant effort has been directed toward developing a COVID‐19 vaccine, which is viewed as the route out of the pandemic. Much of this effort has coalesced around COVAX, the multilateral initiative aimed at accelerating the development of COVID‐19 vaccines, and ensuring they are equitably available in low‐ and middle‐income countries (LMICs). This paper represents the first significant analysis of COVAX, and the extent to which it can be said to have successfully met these aims.

**Methods:**

This paper draws on the publicly available policy documents made available by the COVAX initiatives, as well as position papers and public statements from governments around the world with respect to COVID‐19 vaccines and equitable access. We analyze the academic literature regarding access to vaccines during the H1N1 pandemic. Finally, we consider the WHO Global Allocation System, and its principles, which are intended to guide COVAX vaccine deployment.

**Findings:**

We argue that the funding mechanism deployed by the COVAX Pillar appears to be effective at fostering at‐risk investments in research and development and the production of doses in advance of confirmation of clinical efficacy, but caution that this represents a win‐win situation for vaccine manufacturers, providing them with opportunity to benefit regardless of whether their vaccine candidate ever goes on to gain regulatory approval. We also argue that the success of the COVAX Facility with respect to equitable access to vaccine is likely to be limited, primarily as a result of the prevalence of vaccine nationalism, whereby countries adopt policies which heavily prioritize their own public health needs at the expense of others.

**Conclusions:**

Current efforts through COVAX have greatly accelerated the development of vaccines against COVID‐19, but these benefits are unlikely to flow to LMICs, largely due to the threat of vaccine nationalism.

A vaccine is viewed as the key to bringing about the end of the COVID‐19 pandemic. The sooner a vaccine is available, the sooner the world can begin to escape the acute phase of the pandemic, suppressing mortality and morbidity caused by infection and restoring a degree of normality to social life and the global economy. Not only is global equitable access to a COVID‐19 vaccine an important public health tool, but it is also necessary to ensure that all countries can discharge their human rights obligations.^1^ In an attempt to accelerate the availability of vaccines and other tools to combat COVID‐19, the World Health Organization (WHO) established the Access to COVID‐19 Tools (ACT) Accelerator, a global initiative designed to harbor international cooperation and knowledge regarding the pandemic. Specifically, the ACT‐Accelerator is focused on accelerating development in four areas, or pillars: diagnostics, therapeutics, vaccines (called COVAX), and health systems strengthening.

Although efforts to develop a vaccine are starting to prove successful, with the development of successful candidates from Pfizer, Moderna, and AstraZenica/University of Oxford all receiving emergency regulatory approval in late 2020, key questions remain about which countries will have access to these vaccines, when they will get access, and in what quantities. During the 2009 H1N1 influenza pandemic, procurement of pandemic vaccines was dominated by developed countries, which used advance purchase agreements to reserve doses ahead of production. This severely limited the number of doses available in developing countries.^2^ In addition to accelerating research and development (R&D) through the ACT‐Accelerator, and in an attempt to ensure more equitable access to vaccines for COVID‐19, the Coalition for Epidemic Preparedness Innovations (CEPI); Gavi, the Vaccine Alliance; and the WHO formed the COVAX Facility in early 2020. The COVAX Facility is designed to address the issues encountered by developing countries during the 2009 H1N1 pandemic by using significant advance market commitments to secure access to vaccines on their behalf. It also encourages multilateral cooperation to increase access to vaccines in all participating countries.

This paper argues that the funding mechanism deployed by the COVAX pillar appears to be effective at fostering at‐risk investments in R&D and the production of doses in advance of confirmation of clinical efficacy. Indeed, the development of two vaccine candidates funded by the ACT‐Accelerator is testament to this fact. However, this comes with a caution that the mechanism heavily favors pharmaceutical companies, for which up‐front investment from the COVAX pillar represents a win‐win situation and an opportunity to benefit regardless of whether their vaccine candidate ever goes on to gain regulatory approval. The paper then discusses the COVAX Facility, arguing that, like any multilateral purchasing system, securing a sufficient degree of interest and participation is essential to its success. It argues that the COVAX Facility has so far failed to do this, primarily as a result of the prevalence of vaccine nationalism, whereby countries adopt policies that heavily prioritize their own public health needs at the expense of others, and that its success is therefore likely to be limited. The paper concludes by arguing that the Global Allocation System, designed by the WHO, fails to address the issues experienced by the Vaccine Deployment Initiative during the 2009 H1N1 pandemic, specifically the delays in deploying vaccine in developing countries owing to a lack of preparedness and vaccine utilization infrastructure. It argues that, if these issues are not addressed, the COVAX Facility will fail to secure equitable access to vaccines in developing countries, despite the rapid development of successful vaccine candidates, because the allocation framework will be unable to operate as intended.

## The Access to COVID‐19 Tools Accelerator

As the severity of the SARS‐CoV‐2 virus became apparent in the early part of 2020, focus turned to the rapid development of medical countermeasures to bring about an end to the pandemic. In April, the United Nations General Assembly adopted Resolution 74/274, in which the assembly acknowledged the crucial role of the WHO in coordinating the international response and called on member states to increase R&D funding for tools to combat COVID‐19.^3^ This was followed a few days later by the launch of the WHO's ACT‐Accelerator. The ACT‐Accelerator brings together a number of international organizations and provides a platform for the consolidation of funding efforts and resource sharing. Its goal is to speed up an end to the pandemic by supporting the development and equitable distribution of tools designed to combat COVID‐19.^4^ Work and investment across the four pillars—diagnostics, therapeutics, vaccines, and health system strengthening—is aimed not just at R&D, but also at ensuring the products created are available for equitable distribution in all countries around the world.

The most recent figures show that the ACT‐Accelerator has raised a total of $5.8 billion in funding commitments, but faces an immediate funding gap of $3.7 billion and requires a further $23.7 billion throughout 2021.^5^ The ACT‐Accelerator is a step in the right direction in terms of fostering global cooperation to tackle the pandemic. However, the initiative is yet to secure backing from major powers such as China, India, and Russia.^6^

## The ACT‐Accelerator Vaccines Pillar

Also referred to as the COVAX pillar, the vaccines pillar of the ACT‐Accelerator is convened by CEPI, Gavi, and the WHO and aims to accelerate the development, manufacture, and delivery of COVID‐19 vaccines in all participating countries.^4^ The pillar acts as a pooling mechanism, combining the resources of all participating countries, which will share the risks and benefits associated with investment in vaccines for COVID‐19. The COVAX pillar intends to ensure that the most promising vaccine candidates receive sufficient financial backing, enabling the pillar to assemble a more diverse portfolio of vaccine candidates than any country could do alone.^7^ As noted, the COVAX pillar faces an immediate funding gap of $3.7 billion from 2020, which the United States has indicated it will fill in the short‐term, but does not include the additional $23.7 billion requested to cover funding needs throughout 2021.^5^ As of this writing, it looks unlikely that the pillar will meet its ambitious funding target. It is not clear how detrimental failure to reach this target will be on equitable access to vaccines.

The COVAX pillar set out with the lofty ambition to distribute two billion doses of COVID‐19 vaccine worldwide by the end of 2021.^8^ With this target in mind, the COVAX pillar is focused broadly on achieving three objectives: (1) rapidly accelerating the development of vaccines for COVID‐19 by providing financial backing to a range of promising candidates; (2) using push and pull financing mechanisms to stimulate at‐risk investment in manufacturing capacity; and (3) ensuring equitable access to vaccines around the world, including procuring, allocating, and deploying doses to developing countries through the COVAX Facility (discussed separately). Despite rapid acceleration of vaccine development resulting in the licensure of two vaccines in the COVAX pillar vaccine portfolio in late 2020, as we argue later in the paper, the pillar faces an uphill battle in achieving its goals, particularly in the face of rising vaccine nationalism.

### Accelerating Vaccine Development

The process of developing and gaining regulatory approval for a safe, effective vaccine against a novel disease can typically take well over a decade to complete.^9^ Prior to COVID‐19 the shortest development period for an entirely new vaccine was four years.^10^ Despite this, COVAX set itself the ambitious target of deploying two billion doses before the end of 2021, which required the vaccine development to be accelerated significantly. To achieve this, the COVAX pillar invested $2.4 billion in vaccine R&D, split among a wide portfolio of vaccine candidates to maximize the chances of a successful candidate being developed in the shortest possible time frame.^8^ The emphasis on expediting this process in respect to vaccines for COVID‐19 resulted in pharmaceutical companies experimenting with a range of novel, nontraditional vaccine platforms and technologies, such as mRNA‐ and adenovirus vector–based vaccines.^11^ For example, the AstraZeneca/Oxford candidate is a viral vector adenovirus‐based vaccine,^12^ and two of the COVAX pillar's other portfolio candidates utilize mRNA platforms.^12^ The unprecedented effort mounted by COVAX, CEPI, and governments around the world to accelerate the development of vaccine candidates for COVID‐19 has already borne fruit; two of the vaccine candidates that formed part of the COVAX pillar's R&D portfolio have received licensure in at least one country.

In the first instance, the Moderna vaccine received regulatory approval in the United States and Canada in late December 2020, followed by the European Union in early January 2021. However, despite the fact that the Moderna vaccine was funded as part of the COVAX pillar's R&D portfolio, COVAX has not contracted for the supply of Moderna vaccine. This is likely because distribution of the Moderna vaccine in low‐ to middle‐income countries (LMICs) is complicated by the fact that the vaccine, which uses mRNA technology, needs to be stored at −70 degrees Celsius. In the most recent 2005 estimate, the WHO concluded that up to 50% of vaccines are wasted globally every year, in large part because of lack of temperature control and the logistics to support an unbroken cold chain.^14^ This estimate is based on most vaccines needing to be stored between 2 and 8 degrees Celsius, not −70 degrees Celsius, which presents what currently appears to be insurmountable challenges for rapid rollout of the Moderna vaccine in LMICs.

More encouragingly, the COVAX pillar–funded AstraZeneca/Oxford candidate also received regulatory approval in late 2020, albeit only in the United Kingdom at present. Being an adenovirus vector vaccine, this vaccine represents the most viable vaccine for rollout to LMICs because it can be rapidly manufactured globally, only needs to be stored between 2 and 8 degrees Celsius, and is priced significantly lower than the mRNA vaccines currently licensed.^15^ However, at present, neither of these vaccine candidates has received WHO prequalification or approval from the WHO's Emergency Use Listing (EUL) process, which enables LMICs to expedite their own regulatory approval processes to import and administer the vaccine.

### Manufacturing Capacity and “At‐Risk” Production

Despite the fact that the COVAX pillar has successfully accelerated the vaccine development process and one of the COVAX portfolio candidates has achieved regulatory approval, making the vaccine available in the volumes necessary to reach its goals is a significant challenge to COVAX and its partners. Ensuring adequate and timely manufacturing capacity for COVID‐19 vaccines is essential if COVAX is to meet its target of distributing two billon doses by the end of 2021, a proposition reflected in the $7 billion budgeted for “market preparation and manufacturing.” Of this amount, $6.4 billion was needed before the end of 2020,^8^ a target that was not met.

Vaccine production is complex and expensive, and typically manufacturers do not risk the investment in scaling up production capacity for vaccines without knowing that their candidate works and has been approved for commercial sale. However, given the unique circumstances of COVID‐19, if manufacturers had waited until the efficacy of their candidate was proven before investing in scaling up manufacturing capacity, there would have been further delay in making vaccines available for deployment. The COVAX pillar therefore made an “at‐risk” investment in manufacturing capacity prior to the results of efficacy trials so that doses of COVID‐19 vaccine could be made available as soon as possible.^4^ This was achieved with a combination of push and pull financing mechanisms.^4^ Push financing consisted of direct, at‐risk investment in global vaccine‐manufacturing capacity.^16^ Pull financing was supplied in the form of advance market commitments to purchase substantial volumes of vaccine in the event that a candidate is successful.^16^ This approach was intended to encourage vaccine manufacturers to invest in scaling up manufacturing capacity in two ways: (1) by offering to effectively share the risk of investing in capacity that may never be fully utilized or, more pressingly, be capable of turning a profit; and (2) by offering commitments to purchase a substantial volume of vaccine in the future, which encourages investment in development and manufacturing by effectively guaranteeing future sales. There is some indication that this strategy is working; AstraZeneca started production of its vaccine candidate in June, despite it not being clear at that stage that the vaccine would achieve regulatory approval.^17^ This at‐risk production stems from CEPI investing approximately $383 million, which was used by AstraZeneca to increase manufacturing capacity,^17^ significantly reducing the gap in time between regulatory approval and delivery of doses.

Ultimately, vaccine manufacturers have little to lose under this arrangement. If the candidate is unsuccessful, the investment wasted in producing doses at risk will be significantly lower because the financial risk is being shared with the COVAX pillar. Conversely, if their candidate is successful, manufacturers benefit in two ways. First, the doses produced at risk can be used to begin filling existing purchase orders for vaccine in a shorter than expected time frame. And second, the increased manufacturing capacity at a discounted cost (since at least part of the investment will have been provided by the COVAX pillar) enables them to complete future orders more quickly.

This argument is equally applicable to the R&D side of the COVAX pillar, because any contributions made by the pillar effectively reduce the costs of R&D for the pharmaceutical companies, thereby increasing the potential for profit if the candidate is successful. This arrangement is therefore a win‐win for pharmaceutical companies, because the COVAX pillar bears a significant share of the financial risks associated with failure, leaving the pharmaceutical companies free to profit on the back of any success. In essence, the arrangement privatizes profit and socializes risk. However, it would be unfair to consider this as a failure of the COVAX pillar, because one of its key objectives is to accelerate the development and manufacture of vaccines for COVID‐19, and these arrangements, as much as they stand to benefit pharmaceutical companies, have resulted in the fastest‐ever development of a vaccine for a novel pathogen.

Nevertheless, this leaves open the question of value for money in the development of vaccines for COVID‐19; it may be the case that the amounts that the COVAX pillar and governments around the world have invested in the R&D of the vaccines in their portfolio are substantially more than the costs (actual and opportunity costs) paid by those firms in R&D and expanding manufacturing. If this is the case it raises significant concerns around the price point and profit margins of vaccines in the COVAX portfolio, and much wider concerns regarding how pharmaceutical R&D is incentivized and funded during a health emergency. Ultimately, it is difficult to answer this question due to a lack of transparency in the contractual arrangements between governments and nongovernmental organizations on the one side and the pharmaceutical industry on the other for the development and supply of COVID‐19 vaccines.

Vaccine‐manufacturing capacity is physically and geographically limited, especially with regard to new technological platforms. For example, technologies such as the mRNA technology used by Moderna and Pfizer in their COVID‐19 vaccines are not readily found outside of a small number of high‐income countries (HICs).^18^ With respect to intellectual property (IP) and manufacturing capacity, IP rights are a barrier to expanding manufacturing capacity for many medical products, especially solid‐dose drugs. Were it not for IP rights, stringently enforced by their owners, many of the world's most desperately needed medicines could be easily and cheaply made by generic manufacturers around the world. That is not the case for vaccines; it is not patent protection that is the barrier to introducing generic vaccines, but rather the inaccessibility of knowledge that is not in the public domain and know‐how which is the true barrier to expanded manufacturing capacity for vaccines.^19^ As a result, use of tools such as compulsory licensing of patents (which have proven successful in expanding access to drugs in developing countries) do not represent a viable procurement method to rapidly expand access to a COVID‐19 vaccine.^20^

## The COVAX Facility and Equitable Access

Within the COVAX pillar, which is dedicated to vaccine development, is the COVAX Facility, an initiative concerned specifically with procurement, allocation, and delivery of vaccines for COVID‐19. Participation in the facility is voluntary and not necessarily linked to participation in other parts of the ACT‐Accelerator or the COVAX pillar. The COVAX Facility has entered a range of agreements with manufacturers for the supply of COVID‐19 vaccines, which it believes will be sufficient to reach its target of procuring two billion doses by the end of 2021, approximately half of which will be reserved for deployment in developing countries.^4^ To this end, COVAX reports that it has secured “170 million doses of the AstraZeneca/Oxford candidate via an advance purchase agreement”; 200 million doses (and options for up to 900 million more) of the AstraZeneca/Oxford or Novavax candidates, via “an agreement with the Serum Institute of India”; 500 million doses of the Janssen candidate, via a “memorandum of understanding” with Johnson & Johnson; 200 million doses of the Sanofi/GSK vaccine candidate, via a “statement of intent”; and first “right of refusal for a potential combined total of over 1 billion doses in 2021 of promising vaccine candidates … in the COVAX R&D Portfolio.”^22^

The use of different language to describe these various agreements is indicative of how firm the commitments actually are. “Memorandum of understanding” and “statement of intent” can be taken as synonyms for no formal agreement has yet been reached, but negotiations are underway. It is noteworthy that COVAX differentiates between an “agreement” and an “advance purchase agreement.” As explained earlier, an advance purchase agreement allows a country, or in this case COVAX, to reserve doses of a vaccine prior to licensure or the vaccine being developed, thereby securing priority access as soon as the product becomes available. It is not clear what “an agreement” is in the context of the COVAX Facility, or how this differs from an advance purchase agreement. It is expected that vaccine secured via advance purchase agreements will be supplied before any other agreements (that was certainly the case in previous pandemics^2^), and as we elaborate later in the paper, a significant amount of the AstraZeneca/Oxford vaccine supply has been reserved via bilateral advance purchase agreement by HICs. Therefore, it is likely that the “200 million doses (and options for up to 900 million more) of the AstraZeneca/Oxford” vaccine from the Serum Institute of India will be delivered to COVAX in a slower time scale than those secured via advance purchase agreements by HICs.

By entering advance purchase agreements with manufacturers, the COVAX Facility aims to prevent a scenario in which developing countries are reliant solely on bilaterally donated vaccine to immunize their populations, as was the case during the 2009 H1N1 pandemic.^2^ During the 2009 H1N1 pandemic, developing countries received vaccine much later and in far smaller quantities than developed countries, which were able to procure vaccine using their own advance purchase agreements.^2^ For developed countries, participation in the COVAX Facility represents the opportunity to diversify their procurement strategy for COVID‐19 vaccines, while also supporting access to vaccines in developing countries.^16^ If it is successful, the COVAX Facility has the potential to significantly improve equitable access to COVID‐19 vaccines by expanding access and minimizing delays in their availability in LMICs. If it fails, access to vaccines in developing countries and in countries unable to enter advance purchase agreements will be significantly limited.

The following section explains how the COVAX Facility operates, including the distinction between the self‐funded group (made up primarily of developed countries) and the funded group (made up of developing countries).

### Funding Arrangements

The way in which countries participate in the COVAX Facility depends on which group they fall into, based on their financial resources. HICs and upper middle‐income countries (UMICs) are classed by COVAX as “self‐funded,” whereas low‐income countries (LIC) and LMICs are “funded.”^7^

Self‐funded countries engaging with the facility finance vaccines using their own public finance budgets and are “guaranteed a sufficient number of doses to immunise 20% of their populations.”^7^ These countries provide an up‐front payment and a commitment to purchase their allocated doses through COVAX once they become available. Using these funds, the facility plans to procure approximately 950 million out of the targeted two billion doses. However, the procurement arm of COVAX appears significantly underfunded. Although COVAX reports it “has met its urgent 2020 fundraising target of US$ 2 billion,” it further reports “at least US$ 4.6 billion more is needed in 2021 to procure doses of successful candidates as they come through the portfolio.”^22^ The implication is that unless COVAX receives this significant increase in funding, it will be unable to actively procure doses of vaccine on behalf of its members. Importantly, self‐funded countries are not prohibited from entering bilateral agreements with pharmaceutical companies and are encouraged to use the COVAX Facility as an insurance policy to mitigate against the risk of securing no vaccine from their bilateral agreements, in the event that the candidates are unsuccessful.^21^ However, as we argue in the next section, bilateral advance purchase agreements have undermined the COVAX Facility by increasing competition for a limited supply of vaccine, thereby reducing the number of doses available for timely procurement by the facility.

In contrast, the application process for funded countries required no up‐front payment. The participation of funded countries has been supported by the Gavi COVAX Advance Market Commitment (AMC), a financing instrument designed to support the procurement and delivery of COVID‐19 vaccines for developing countries.^21^ The premise of the COVAX Facility is to ensure equitable access to vaccines for COVID‐19 by pooling international resources, enabling investment and advance procurement from a range of vaccine candidates, and sharing the potential risks and benefits, while mitigating against the risks associated with countries going it alone in vaccine procurement. The world cooperates to develop the vaccine and to procure it, meaning that once doses become available, they can be distributed equitably across the world.

However, the success of the facility is dependent on securing sufficient backing from the international community, which has not been forthcoming. This is important for two main reasons: first, as just discussed, significant financial backing is needed to ensure the facility can meet its targets for procurement; and second, a more cohesive international approach to procurement benefits the facility by reducing competition for doses in the early stages when availability will be limited.

In addition to chronic underfunding, a significant challenge to the success of COVAX is the reemergence of vaccine nationalism during COVID‐19, whereby developed countries prioritize conducting their own bilateral agreements with pharmaceutical companies over the multilateral procurement system.^23^ Such behavior follows a pattern of nationalist actions taken by countries throughout the pandemic, including temporary restrictions on travel and trade.^24^

### Participation in the Facility and the Threat of Vaccine Nationalism

The main threat to the facility securing sufficient participation is the reemergence of vaccine nationalism, whereby countries prioritize conducting their own bilateral advance purchase agreements with vaccine manufacturers over participation in multilateral initiatives such as the COVAX Facility.^23^ A shift toward vaccine nationalism is evident in the current pandemic. While 69 countries, plus the EU trading bloc, have formally joined COVAX, and a further 86 have submitted an expression of interest in doing so, there are some notable absentees.^21^ China and Russia appear to have wholly rejected the COVAX Facility in favor of pursuing their own bilateral agreements with vaccine manufacturers. Further, a significant number of HICs have completed their own bilateral agreements in addition to considering participating in the facility.

One example of this threat is the European Union. EU officials initially advised member states against joining the facility because they believed it would lead to “higher prices and later supplies.” The EU officials also warned that participating in the facility may be incompatible with an exclusivity clause signed by its member states.^21^ This decision was ultimately reversed in September 2020, and the EU joined COVAX. However, despite this purported commitment to COVAX, EU member states will also be able to benefit from advance purchase agreements negotiated by the European Commission using its 2.7 billion‐euro Emergency Support Instrument,^25^ as well as those negotiated by the Inclusive Vaccine Alliance (IVA).^26^ The IVA is an alliance consisting of France, Germany, Italy, and the Netherlands and has negotiated an agreement with AstraZeneca for the supply of 300 million doses, which will be distributed proportionately among EU member states that wish to participate.^27^ This deal has now been ratified by the commission, which has also concluded agreements with five other companies to secure at least 1.9 billion doses of vaccine through advance purchase agreements. While the situation with EU member states is in fact an example of multilateral cooperation to procure vaccines for COVID‐19 at scale, the focus of both the commission and the IVA is to secure equitable access to vaccines within the EU, rather than in all countries around the world.

The EU countries are not alone in their decision to pursue advance purchase agreements outside of the COVAX Facility. The United Kingdom has entered seven advance purchase agreements on its own, potentially securing access to approximately 357 million doses of vaccine.^29^ Canada has also announced deals with Pfizer and Moderna for “millions of doses” of their vaccine candidates.^30^ Both Canada and the United Kingdom submitted a nonbinding expression of interest in joining the facility in July 2020,^31^ but the extent of their own bilateral agreements, particularly in the case of the United Kingdom, suggests that they are unlikely to fully commit to, or be reliant on, participating in the facility to meet their procurement needs. Although the COVAX Facility has been clear that countries conducting their own bilateral agreements can use the facility as an “insurance policy,”^21^ it is difficult to see what more the facility is capable of offering countries such as Canada and the United Kingdom that already have advance purchase agreements in place with all of the leading candidates. To appeal to countries in this position, the facility needs to consider how it can offer a more diverse portfolio of vaccine candidates, while still ensuring that it reaches agreements with the leading candidates so that the other participating countries, which are unable to enter their own bilateral agreements, can still benefit from them.

Under the leadership of President Trump, the United States expressed no interest in joining the COVAX Facility and was clear from early in the pandemic that the ambitious Operation Warp Speed was focused on fulfilling America's needs first, before going on to assist the rest of the world.^32^ As part of Operation Warp Speed, the United States has invested in a range of vaccine candidates. These include a deal with Pfizer and BioNTech to produce an initial 100 million doses, a deal with AstraZeneca for 300 million doses, and an agreement with Novavax for “at least” 100 million doses.^32^ Under the Biden administration, the United States has made significant funding commitments to COVAX, although it continues to agressively pursue its own bilateral agreements over procurement through COVAX.^33^

In addition to the United States, many developed countries have decided to make monetary donations to the COVAX AMC, but still have not yet joined the COVAX Facility as a participating country. This is a further indication that the COVAX Facility has failed to win the support of these countries. The United Kingdom, Canada, Germany, Italy, and Sweden have pledged donations totalling approximately $960 million,^34^ but each has only given “non‐binding confirmations of intent to participate in the COVAX Facility.” These pledges suggest that countries are willing to fund the COVAX AMC as a mechanism for promoting equitable access in developing countries, but have reservations about participating in the COVAX Facility as the mechanism through which to procure their own vaccines. This reluctance to rely on the COVAX Facility for procurement of COVID‐19 vaccines, coupled with the willingness to provide funds directly to the COVAX AMC while pursuing bilateral advance purchase agreements, suggests that other countries share the fears expressed by the EU that the facility will fail to deliver vaccines on the correct time scale and/or at the right price point. This half‐in, half‐out approach to multilateral cooperation can only be detrimental to the COVAX Facility in the long term, and it reinforces fears (discussed next) that the facility will begin to receive doses only after developed countries have started to receive their supplies.

This shift toward vaccine nationalism and the fragmentation of the global procurement landscape will have a detrimental impact on the efforts of the COVAX Facility and, in turn, equitable access to vaccines for COVID‐19, particularly in developing countries. Given the proliferation of bilateral advance purchase agreements and underfunding of the COVAX Facility, some HICs (or alliances) will receive priority access over others. At this stage, it does not look as though the COVAX Facility will receive priority over countries that have concluded bilateral purchase agreements. For example, both the United Kingdom and the facility have reached agreements with AstraZeneca, but the United Kingdom has already begun to receive its allocation, whereas the COVAX Facility has not, and there is no clear indication of when rollout in COVAX participating countries will begin.^4^ Moreover, given the number of doses secured bilaterally by HICs compared to COVAX, it is clear that COVAX‐reliant countries will receive a significantly smaller number of doses than HICs with bilateral agreements in place. The issue of priority access is significant, because countries capable of conducting their own bilateral agreements are unlikely to engage fully with the COVAX Facility if doing so will result in delays in receiving doses of vaccine. Indeed, part of the EU's initial decision not to engage with the facility was predicated on the idea that the EU countries are aiming to start receiving doses in early 2021 and officials believe this schedule is “not feasible” through the COVAX Facility.^35^ Hence, the EU continues to aggressively pursue bilateral agreements, despite “committing to COVAX.”

If countries that have conducted bilateral advance purchase agreements receive priority access to vaccines for COVID‐19, it will be at the expense of the COVAX Facility. In turn, this will hinder timely access to vaccines in developing countries because individual countries are primarily concerned with fulfilling their domestic needs, rather than those of all countries around the world. As developing countries are unlikely to have the financial resources to commit to advance purchase agreements themselves,^2^ it follows that any delay in the COVAX Facility receiving vaccines will disproportionality affect developing countries. Ultimately, unless a significant majority of countries are exclusively engaged with the facility, it becomes just another competitor in the global market for access to vaccines for COVID‐19, albeit one focused on securing global equitable access, rather than fulfilling domestic need. Unless it can get to the front of the queue—which current evidence suggests is not the case—the facility, and the developing countries reliant on it, will experience significant delays in securing doses, thereby limiting equitable access to vaccines.

### Allocation and Delivery

When the COVAX Facility begins to receive doses, precisely how they will be allocated to participating countries depends on whether they are self‐funded or COVAX AMC supported (i.e., funded). As stated earlier, self‐funded countries participating in the facility are “guaranteed enough doses to immunise 20% of their population, with doses to be distributed equitably as they become available.”^16^ An equal allocation of doses will be distributed among the funded countries, but how these are allocated will be governed by the WHO's Global Allocation Framework.^16^

The current proposal would see vaccine delivered in two phases. During phase 1, countries will receive doses proportionally based on their total population.^36^ Allocation during this phase will be focused initially on supplying a sufficient number of doses to immunize health care and social workers, for which it is estimated countries will need a quantity of doses equivalent to 3% of population coverage.^36^ Following this, focus will shift to immunizing high‐risk adults, including the elderly and adults with comorbidities, until countries have received enough doses to immunize up to 20% of their populations.^36^ Phase 2 will allow for coverage beyond the 20% mark and doses will be allocated based on country need, vulnerability, and the relative threat of COVID‐19 within the population.^36^ However, the Global Allocation Framework operates on the fundamental principle that all countries should receive doses at the same rate to the extent possible.^36^

Several practical and logistical issues associated with transporting and deploying vaccines make the WHO's commitment to delivering doses to funded countries at the same rate difficult to fulfill. For example, transporting vaccines often requires sufficient cold‐chain infrastructure.^37^ Although the vaccine developed by AstraZeneca will be able to leverage the standard cold‐chain infrastructure, which is currently widely available in developing countries,^38^ the vaccines utilizing mRNA platforms require significantly more sophisticated infrastructure. In addition, global distribution of vaccines necessitates sophisticated storage facilities that comply with Good Manufacturing Practices (GMP) and are operated by appropriately trained employees.^37^ Although the COVAX Facility and in particular Gavi have committed to supporting development of cold‐chain infrastructure and readiness of supply chains in developing countries,^16^ the reality is that some countries will be better prepared than others and several WHO member states have expressed concern about how countries will be prioritized during phase 1 when vaccine supplies are likely to be severely constrained.^36^

During the 2009 H1N1 influenza pandemic, the WHO established the Vaccine Deployment Initiative (VDI) to manage the allocation and delivery of donated influenza vaccines in developing countries. Countries were prioritized based on their ability to complete the application process established by the VDI. This involved reaching agreement on legal issues, such as liability waivers, and completing a comprehensive “national deployment plan,” demonstrating the countries’ capacity to utilize doses effectively.^2^ Prioritizing countries that had sufficient vaccine deployment infrastructure reduced the number of doses that may have been wasted. However, many countries struggled to rapidly produce a national deployment plan, resulting in significant delays in doses being delivered. In the African region, the average time between a country starting the application process and receiving its first doses was 261 days.^2^ In many cases, this meant that doses arrived only after the acute phase of the pandemic had passed, at which point they were less effective.^39^

Given that supply of vaccines for COVID‐19 will also be limited, it may be necessary for the COVAX Facility to adopt a similar mechanism that prioritizes countries capable of using vaccine in order to prevent doses going to waste. The mechanism used during the 2009 H1N1 pandemic sought to do this by prioritizing countries that were able to satisfy the application criteria quickly; these were typically the more advanced countries, which already had some of the necessary infrastructure in place or were able to make arrangements relatively quickly. The system therefore penalized countries that were not able to satisfy the application process quickly, typically the poorest countries and those that required significant assistance from the WHO to complete the national deployment plan.^39^ A similar mechanism therefore risks penalizing the very poorest countries lacking sophisticated vaccine utilization infrastructure and would frustrate the fundamental principle of the Global Allocation Framework that countries should receive doses at the same rate.

The WHO considered the issue of what happens if a country is “not ready” to receive vaccine during a member state briefing on August 13, 2020.^40^ Countries that have fallen behind in terms of allocation will be caught up by receiving larger doses in subsequent delivery cycles. For example, in month 1, countries might receive enough doses to cover 3% of their populations, followed by a further delivery in month 2 to take them to 7% population coverage. If a country is not ready to receive doses during month 1, it will then receive enough doses to reach 7% population coverage during month 2.^40^ Although this approach has the potential to ensure that countries receive a proportional number of doses relative to their population, it does not address the fact that countries lacking the requisite vaccine infrastructure will still receive doses later than others. This approach also assumes that the COVAX Facility will have access to a sufficient number of doses to make significantly larger deliveries to some countries in later delivery cycles, something that is impossible to confirm. This solution attempts to sidestep the issues arising from a lack of vaccine infrastructure, rather than tackling them directly, and therefore fails to address the issues experienced by the VDI during the 2009 H1N1 pandemic.

To avoid inequity of vaccine distribution on the same scale as during the 2009 H1N1 pandemic, the COVAX Facility should invest heavily in scaling up the necessary infrastructure in funded countries so that doses can begin to be deployed in all countries, at the same rate, as soon as possible. This is especially required with respect to the mRNA vaccine candidates, which need to be kept at −70 degrees Celsius; otherwise, these vaccines, if they are licensed, will not be able to be rolled out in developing countries, creating an inequity in product availability between developed and developing countries. To date, COVAX has not contracted for either of the mRNA vaccines that have received licensure (i.e., the Moderna and Pfizer vaccines). However, as part of the COVAX pillar's R&D portfolio, the organization has invested in bringing the Moderna candidate to market. This raises questions about the disconnect between COVAX as a mechanism to incentivize R&D for COVID‐19 vaccines and its role as procurement agent for developing countries.

Widespread improvements in vaccine infrastructure in funded countries would reduce the need for an allocation mechanism that prioritizes countries based on their readiness to utilize vaccine, allowing for more equitable distribution of doses in line with the fundamental goal of the Global Allocation Framework. In the long term, this investment would improve the readiness of the funded countries to deal with future pandemics, as well as routine immunization campaigns. Therefore, just as the facility aims to encourage the manufacturing of vaccines at risk, it should also encourage and support at‐risk development in vaccine delivery infrastructure. Without this, the significant funds being invested in the accelerated development and manufacturing process will benefit only those countries that already have sufficient infrastructure in place.

## Conclusion

The COVAX Facility represents a significant attempt to facilitate multilateral cooperation to procure vaccines for COVID‐19 and to distribute those vaccines equitably, in all countries around the world. This paper has explained how the facility intends to meet its ambitious target of distributing two billion doses of vaccine across all participating countries before the end of 2021, exploring the issues with its at‐risk funding strategy and the barriers it faces in fostering such a significant international collaboration.

Three main arguments have been put forth. The first concerns the facility's at‐risk funding strategy, in particular the way in which it creates a win‐win situation for pharmaceutical companies. Increasing vaccine‐manufacturing capacity and accelerating the availability of vaccines for COVID‐19 should undoubtedly be considered a public good, but the risks associated with the facility's at‐risk financing strategy should not be underestimated. Indeed, given the fact that two vaccine candidates in the COVAX pillar's R&D portfolio have received emergency licensure suggests that the COVAX pillar has been successful in accelerating the development of COVID‐19 vaccines. However, the same cannot be said of COVAX as a procurement tool for LICs and LMICs.

The second argument is that the prevalence of vaccine nationalism appears to be limiting the participation of some of the world's wealthiest countries in COVAX. These countries have pursued bilateral advance purchase agreements with the vaccine manufacturers, placing those countries in direct competition with the COVAX Facility for doses when they become available. This act of countries hedging their bets represents an existential threat to the facility and puts its mission in peril.

The final argument is that the current Global Allocation Framework, as envisioned by the WHO, will be insufficient in two regards: (1) in facilitating the facility's two billion‐dose target; and (2) in abiding by its own “fundamental principle” that countries will receive doses at the same rate. The reality is that the facility will be unable to deliver doses to countries at the same rate because not all countries have in place the necessary infrastructure to deploy and utilize doses effectively. This is an issue that hampered the operation of the VDI during the 2009 H1N1 pandemic, and it is one that, we argue, should receive urgent attention in the current pandemic. Unless the disparity in vaccine deployment infrastructure is addressed, the facility's efforts will benefit only wealthier countries that already have sufficient infrastructure in place, and access to vaccines for COVID‐19 in developing countries will be delayed.

*Funding/Support*: This work was supported by a grant from the Arts and Humanities Research Council of the UK (Grant number AH/V006924/1).

*Conflict of Interest Disclosure*: Both authors completed the ICMJE Form for Disclosure of Potential Conflicts of Interest. No conflicts were reported.
